# Revolutionizing Oral Cancer Detection: An Approach Using Aquila and Gorilla Algorithms Optimized Transfer Learning-Based CNNs

**DOI:** 10.3390/biomimetics8060499

**Published:** 2023-10-19

**Authors:** Mahmoud Badawy, Hossam Magdy Balaha, Ahmed S. Maklad, Abdulqader M. Almars, Mostafa A. Elhosseini

**Affiliations:** 1 Department of Computer Science and Informatics, Applied College, Taibah University, Al Madinah Al Munawwarah 41461, Saudi Arabia; 2 Department of Computers and Control Systems Engineering, Faculty of Engineering, Mansoura University, Mansoura 35516, Egyptmelhosseini@mans.edu.eg (M.A.E.); 3 Department of Bioengineering, Speed School of Engineering, University of Louisville, Louisville, KY 40208, USA; 4 College of Computer Science and Engineering, Taibah University, Yanbu 46421, Saudi Arabia; amaklad@taibahu.edu.sa (A.S.M.); amars@taibahu.edu.sa (A.M.A.); 5 Information Systems Department, Faculty of Computers and Artificial Intelligence, Beni-Suef University, Beni-Suif 62521, Egypt

**Keywords:** classification, convolutional neural network (CNN), deep learning (DL), Gorilla Troops Optimizer (GTO)

## Abstract

The early detection of oral cancer is pivotal for improving patient survival rates. However, the high cost of manual initial screenings poses a challenge, especially in resource-limited settings. Deep learning offers an enticing solution by enabling automated and cost-effective screening. This study introduces a groundbreaking empirical framework designed to revolutionize the accurate and automatic classification of oral cancer using microscopic histopathology slide images. This innovative system capitalizes on the power of convolutional neural networks (CNNs), strengthened by the synergy of transfer learning (TL), and further fine-tuned using the novel Aquila Optimizer (AO) and Gorilla Troops Optimizer (GTO), two cutting-edge metaheuristic optimization algorithms. This integration is a novel approach, addressing bias and unpredictability issues commonly encountered in the preprocessing and optimization phases. In the experiments, the capabilities of well-established pre-trained TL models, including VGG19, VGG16, MobileNet, MobileNetV3Small, MobileNetV2, MobileNetV3Large, NASNetMobile, and DenseNet201, all initialized with ’ImageNet’ weights, were harnessed. The experimental dataset consisted of the Histopathologic Oral Cancer Detection dataset, which includes a ’normal’ class with 2494 images and an ’OSCC’ (oral squamous cell carcinoma) class with 2698 images. The results reveal a remarkable performance distinction between the AO and GTO, with the AO consistently outperforming the GTO across all models except for the Xception model. The DenseNet201 model stands out as the most accurate, achieving an astounding average accuracy rate of 99.25% with the AO and 97.27% with the GTO. This innovative framework signifies a significant leap forward in automating oral cancer detection, showcasing the tremendous potential of applying optimized deep learning models in the realm of healthcare diagnostics. The integration of the AO and GTO in our CNN-based system not only pushes the boundaries of classification accuracy but also underscores the transformative impact of metaheuristic optimization techniques in the field of medical image analysis.

## 1. Introduction

Cancers are a group of noncommunicable diseases that can occur almost anywhere in the human body [1]. They are characterized by unregulated cell growth and invasion into neighboring tissues, organs, and other anatomical sites. Among all causes of death, the World Health Organization (WHO) ranks cancer as the second leading killer worldwide. In 2020, there were 19.3 million new cases and 9.96 million deaths [2]. Unfortunately, most cancers continue to pose difficulties regarding early detection, treatment, and prognosis [3].

Early cancer detection improves curability, resulting in significantly less morbidity and mortality than if cancers are detected at more advanced stages [4]. In recent years, medical imaging techniques have played a crucial role in cancer assessment because they provide detailed visualization of the human body’s internal structures, which aids in cancer diagnosis and treatment [5]. In addition, accurate cancer susceptibility, recurrence, and survival predictions are essential to increase patients’ survival rates.

**Figure 1:**
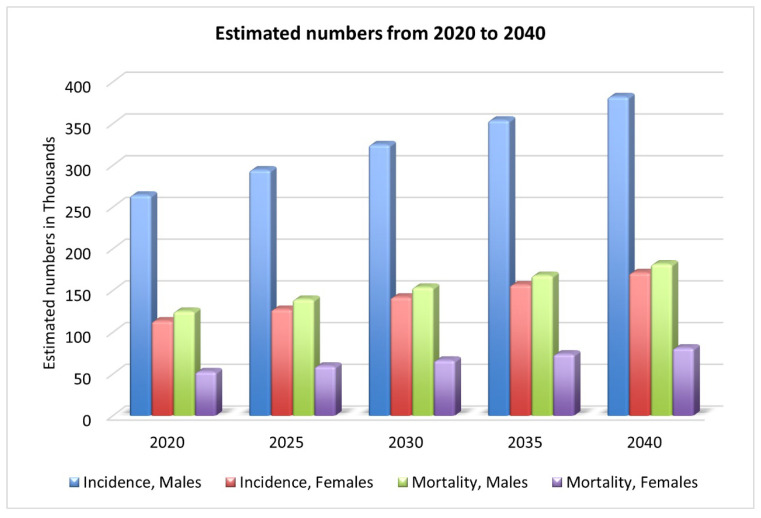
Estimated new cases and deaths from 2020 to 2040 [2].

Oral cancer is a complex, widespread malignancy, reported as the sixth most diagnosed cancer [4]. There were 377,713 new lip and oral cavity cancer cases in 2020, with 177,757 deaths as a result [2]. As shown in Figure 1, by 2030, there will be an estimated 467k new cases and 220k deaths [2]. One of the most lethal diseases of the head and neck is oral cancer, characterized by a wide range of behavior patterns, a high recurrence rate, and an increasing incidence [6]. Comorbidities such as speech impairment, oral pain, malnutrition, dysphagia, and lack of appetite are also common among people with oral cancer, and they contribute to the poor health-related quality of life these patients experience [1]. Oral squamous cell carcinomas (OSCCs) account for more than 90% of all cases of oral cancer [7]; however, only 70% of patients will be alive after five years [1] with this aggressive form of cancer. While lymphoma and leukemia are the most common types of cancer in KSA, OC is the third most common [8].

**Figure 2:**
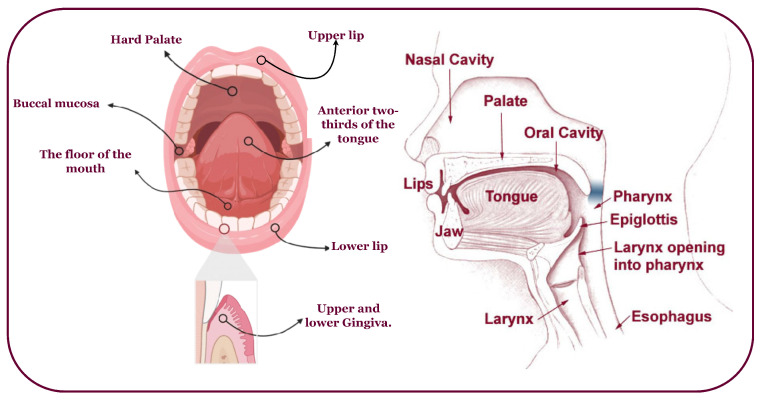
An overview of the head, neck, and possible OC-infected subsites.

Oral cancer predominantly affects the head, neck, and various subsites (Figure 2) [9,10]. It often arises from oral lesions and can potentially spread to other body parts [11]. While advancements in treatment, including chemoradiation, radiation therapy, immunotherapy, and anticancer treatments, have improved, the survival rate remains at 40% to 50% [12]. Early diagnosis and tailored treatment selection are imperative to enhance patient outcomes. Unfortunately, most oral cancer cases are detected late, with early lesions often asymptomatic and benign, making clinical diagnosis challenging [4]. Overcoming obstacles like low awareness, limited screening, and delayed specialist consultation is crucial to prevent misdiagnosis, disease progression, and decreased survival.

The early detection of oral cancer improves patient survival rates [13] and can impact the outcomes for individuals with oral cancer as follows:

- More effective treatment options: Treatment options are more effective when oral cancer is detected early. Surgery, radiation therapy, and chemotherapy are common treatments for oral cancer, and they are most successful when the cancer is localized and has not spread to nearby tissues or lymph nodes.
- Higher cure rates: Early-stage oral cancer is often curable. Patients diagnosed at an early stage have a significantly higher chance of being cured than those diagnosed at an advanced stage, when the cancer has already spread to other body parts.
- Preservation of function and appearance: Early detection may allow less aggressive treatments to preserve important functions such as speech, swallowing, and chewing. It can also help in preserving the patient’s facial appearance.
- Reduced morbidity: Advanced oral cancer can lead to significant morbidity, including disfigurement and difficulty in eating and speaking. Early detection can reduce the extent of surgery required and the associated complications, leading to a better quality of life for the patient.
- Lower healthcare costs: Treating oral cancer at an advanced stage typically involves more extensive and costly interventions. Early detection can lead to less aggressive treatments, shorter hospital stays, and reduced healthcare expenses.
- Improved quality of life: Early detection increases the chances of survival and improves the patient’s overall quality of life. Patients diagnosed and treated at an early stage generally experience fewer side effects from treatment and a faster recovery.

**Figure 3:**
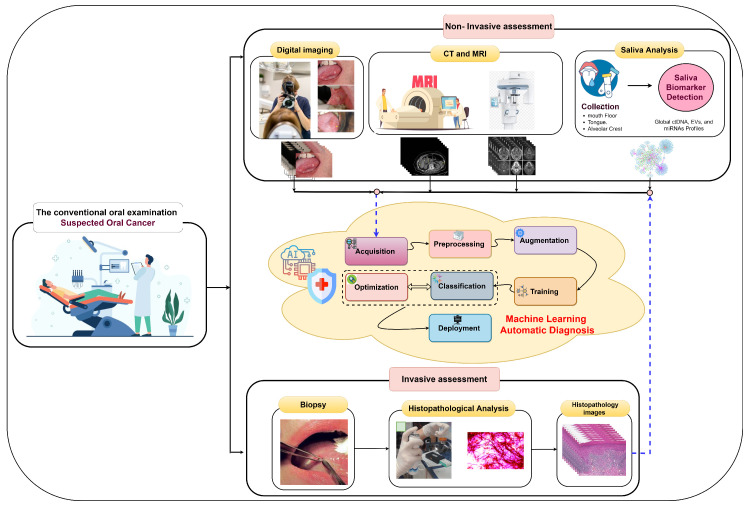
Invasive assessment and non-invasive assessment of OSCC.

Early oral cancer detection can reduce the death rate by 70% [14], underscoring the importance of precise histopathological identification and accurate early detection for informed treatment decisions and improved survival. In the early stages, oral tumors often lack symptoms and manifest as erytholeuko-plastic lesions, including white patches (leukoplakia) or red patches (erythroplakia) [9]. The process of oral tumor identification involves several stages, as illustrated in Figure 3. It begins with conventional oral examinations conducted by dentists and specialists during routine check-ups. Subsequently, two diagnostic approaches are employed. The first is non-invasive and includes digital imaging, biomarker detection in saliva, and medical imaging techniques like computed tomography (CT) and magnetic resonance imaging (MRI) [15]. Pathologists or computer-aided systems can perform non-invasive assessments. The second approach is invasive and entails a tissue biopsy for microscopic analysis. Histology grading is used to classify cancer cells based on tissue abnormalities, focusing on architectural differences and keratin pearls [16]. High-throughput microscopy techniques, such as ex vivo fluorescent confocal microscopy (FCM), can also be employed.

In the invasive assessment, tissue samples are extracted following a clinical examination to confirm the disease’s presence through histological processes. These collected samples are processed, embedded in paraffin blocks, and then sectioned. Various tissue components are stained with different dyes for examination under optical magnification, commonly using hematoxylin and eosin (H&E) staining [1]. Diagnosing oral lesions relies on complex and expensive microscopic examination to detect cyto-histopathological abnormalities by analyzing tissue characteristics [17]. Histopathological images contain valuable phenotypic information for disease management and patient survival [18]. However, this gold standard approach is demanding, requiring experienced pathologists to annotate structures and morphological features on numerous tissue sections, impacting examination accuracy. Given the limitations of current approaches, there is a need for more accurate early screening methods for oral cancer, emphasizing the importance of precise histopathological identification in disease estimation and prognosis.

The rising prevalence of several diseases has forced medical experts to turn to technological aid [19]. In this vein, increasing diagnostic and prognosis accuracy may aid doctors in providing more precise care [6]. Furthermore, whole-slide images (WSIs) enable digital image production from whole-tissue slides at high resolution [18]. However, these microscopic imaging techniques produce unlabeled large-sized microscopic images containing spatial information, cell interactions, and many objects [20]. Manual screenings for oral cancer, while valuable, come with several challenges and limitations, such as (i) subjectivity: manual screenings heavily rely on the subjective judgment and experience of healthcare professionals, such as dentists and oral surgeons. Variability in interpretation can lead to inconsistencies in detecting abnormalities. (ii) Late detection: in some cases, oral cancer may not exhibit visible or palpable symptoms until it reaches an advanced stage. This means that even skilled professionals may miss early signs of cancer during routine screenings. (iii) Patient cooperation: successful manual screenings depend on patients’ ability to cooperate by opening their mouths and remaining fully still. This can be challenging, especially for anxious patients with limited mobility or cognitive impairments. (iv) False positives and negatives: manual screenings can result in false positives (identifying a benign condition as cancerous) or false negatives (missing cancerous lesions). These errors can lead to unnecessary anxiety and additional testing or delayed diagnosis. (v) Time consuming: manual screenings can be time consuming, particularly in busy clinical settings. This may lead to rushed examinations or reduced thoroughness. These challenges can impact the accuracy and effectiveness of early detection efforts. Thus, an automated system is necessary to augment pathologists’ tasks.

Pathomics, integrating machine learning and digital pathology, aims to enhance prognostication. It can analyze a wide range of whole-slide image (WSI) data to generate quantitative features characterizing tissue sample phenotypes [21]. Machine learning has emerged as a promising approach in oncology, supporting disease prevention, accurate diagnoses, treatment decisions, and patient care [19]. This technology effectively analyzes medical images, including lesions and pathologies, facilitating early and precise diagnosis based on macroscopic photographs.

The digitalization of histopathology and pathomics has created a promising field that can transform medical and surgical pathology [20]. Machine learning, particularly deep learning, offers an opportunity to automate feature extraction and classification for early malignancy screening [22]. Despite challenges like limited datasets, heterogeneity, and computational complexity, this study is motivated by several factors: (i) automating time-consuming tasks in visual tissue slide examination can aid pathologists [23]. (ii) Precise automated classification is crucial for early tumor detection. (iii) Deep learning impacts medical diagnosis. (iv) It assists medical professionals in treatment planning; and (v) optimization processes are essential for model design and hyperparameter tuning.

The research focuses on the challenging issue of oral cancer, a complex malignancy with a high incidence rate. Despite remarkable treatment strategy advancements, oral cancer’s survival rate remains distressingly low. Late-stage diagnosis is a primary contributing factor to this unfortunate reality. Early detection is pivotal in enhancing patient outcomes, as it directly correlates with reduced morbidity and mortality rates. Regrettably, early-stage oral cancer lesions often remain asymptomatic, posing significant diagnostic challenges. The current diagnostic methods, which heavily rely on labor-intensive and time-consuming histopathological examination by experienced pathologists, have demonstrated limitations. Therefore, a pressing need exists for developing more reliable and efficient early oral cancer detection screening methods. With this context in mind, the primary research objectives of this study are outlined as follows:

- Development of an automated oral cancer classification model: The foremost goal is to create an innovative model for the automated classification of oral cancer, employing cutting-edge deep learning techniques, specifically, convolutional neural networks (CNNs). This framework will be designed to be adaptable, alleviating the necessity for the manual assignment of hyperparameters and ensuring the automation of the classification process.
- Leveraging transfer learning for enhanced efficiency: Harness the power of transfer learning (TL) to enhance the efficiency and effectiveness of oral cancer classification significantly. This involves capitalizing on pre-trained CNN models to improve the accuracy of our classification system.
- Optimization with Aquila and Gorilla Optimizer algorithms: Investigate the utilization of the Aquila and Gorilla Optimizers. These algorithms will play a pivotal role in optimizing the performance of both the CNNs and the TL process. The aim is to explore the possibilities of improving the classification accuracy. Furthermore, conduct a comparative analysis pitting the Aquila and Gorilla Optimizers against other nature-inspired algorithms to ascertain which optimization approach yields the superior results within the context of oral cancer classification.

In summary, this research addresses the pressing issue of early oral cancer detection through a multifaceted approach that includes automated classification models, transfer learning, and advanced optimization algorithms. The following points summarize the current study’s contributions:

- An innovative model for classifying oral cancer, built upon pre-trained CNNs.
- The fusion of deep learning CNNs with the Aquila and Gorilla Optimizers demonstrates their efficiency in oral cancer classification.
- A comprehensive optimization of each pre-trained model’s performance through meticulous adjustments to the CNN and TL hyperparameters facilitated by the Aquila and Gorilla Optimizers.
- The introduction of an adaptable framework that eliminates the need for manual hyperparameter assignment.
- A thorough comparative analysis between the two optimization algorithms, Aquila and Gorilla.
- Promising outcomes in classification performance, as substantiated by standard performance metrics.

The remaining sections of the paper are as follows: Section 2 presents an overview of deep learning, metaheuristic optimization, and the AO and GTO algorithms. The related research is set out in Section 3. Section 4 presents the strategy and framework that have been proposed. The experimental results and comparisons with state-of-the-art techniques are discussed in Section 5. Finally, Section 6 presents the study’s conclusions.

## 2. Background

Deep learning (DL) is a subset of artificial intelligence that mimics brain functions in data processing and decision making. The potential impact of applying optimized deep learning (DL) models in healthcare diagnostics is profound and far-reaching. Optimized DL models can analyze medical images, such as X-rays, MRIs, CT scans, and histopathology slides, with unprecedented accuracy. This enables the early detection of diseases like cancer, cardiovascular issues, and neurological disorders, increasing the chances of successful treatment and improved patient outcomes. Further, DL models can assist healthcare professionals in making more accurate diagnoses. They can help identify subtle patterns or anomalies that might be missed by human observers, reducing diagnostic errors and ensuring that patients receive appropriate care. They can analyze large datasets to predict disease outbreaks, patient readmissions, and healthcare resource utilization.

Optimized models can analyze a patient’s medical history, genetic data, and other relevant information to recommend personalized treatment plans. This tailoring of treatment can lead to more effective therapies with fewer side effects. Additionally, DL models can automate routine tasks, such as triage, data entry, and medical image analysis. They can accelerate discovery by predicting potential drug candidates, simulating molecular interactions, and identifying disease biomarkers.

While there is an initial investment in developing and optimizing DL models, they can ultimately lead to cost savings in healthcare. Early disease detection, reduced hospitalizations, and more efficient resource allocation can lower healthcare costs in the long run. Moreover, DL models can continuously learn and adapt to new data and research findings. As more patient data becomes available and medical knowledge advances, these models can improve diagnostic accuracy and treatment recommendations. DL models can also be integrated into telemedicine platforms, enabling remote diagnosis and consultation.

Deep learning can address several challenges associated with manual, oral cancer screenings [24]. Deep learning can help to overcome these challenges in the following ways:

- Automation: Deep learning models can automatically analyze medical images without human interpretation, such as oral cavity photos or radiographs. This automation can increase the efficiency of screenings and reduce the burden on healthcare professionals.
- Consistency: Deep learning models provide consistent and objective results, reducing the variability introduced by different healthcare providers. This consistency can lead to more reliable detection of abnormalities.
- Early detection: Deep learning algorithms can detect subtle and early signs of oral cancer that the human eye might miss. They can identify irregular patterns, shapes, and color changes in images indicative of precancerous or cancerous lesions.
- Enhanced visualization: Deep learning can enhance the visualization of challenging areas in the oral cavity, such as the base of the tongue or tonsils, by processing images to highlight potential abnormalities or fusing information from multiple imaging modalities.
- Reduced false positives and negatives: With proper training and validation, deep learning models can significantly reduce the occurrence of false positives and false negatives in oral cancer screenings, leading to more accurate results and reducing patient anxiety.
- Continuous learning: Deep learning models can continuously learn and adapt from new data, allowing them to improve over time as more cases are analyzed. This adaptability can keep the model up to date with the latest information and detection techniques.
- Speed: Deep learning algorithms can quickly process large medical image datasets, leading to faster screenings and potentially earlier diagnosis.
- Risk stratification: Deep learning can help to stratify patients into different risk categories based on the severity of detected abnormalities, allowing healthcare providers to prioritize follow-up care for high-risk individuals.

Deep learning uses multiple nonlinear layers to extract features. Convolutional neural networks (CNNs), a DL subcategory, are commonly used for visual image analysis with minimal preprocessing. CNNs were introduced by LeCun et al. in 1998 for document identification [25]. In recent years, medical professionals have shown increasing interest in using machine learning for diagnostics [25]. DL’s potential is promising, but demands large datasets and it traditionally operates in isolation.

Using pre-trained networks like AlexNet for various tasks, transfer learning breaks this isolation paradigm, enabling knowledge transfer between tasks. Transfer learning adapts existing knowledge to new domains, avoiding the need to start from scratch when learning something new. It involves using components from one model to create a model for a different purpose, often incorporating additional training data and neural layers [26,27]. Transfer learning plays a crucial role in enhancing the accuracy of the classification system in several ways:

- Feature extraction: Transfer learning leverages pre-trained deep learning models (e.g., DenseNet201, VGG16, Xception) trained on large and diverse datasets, such as ImageNet, for general image recognition tasks. These models have learned to extract valuable hierarchical features from images, which are useful for various classification tasks. Instead of training a deep model from scratch, transfer learning allows these pre-trained models to be used as feature extractors.
- Reduced data requirements: Training deep neural networks from scratch often requires a vast amount of labeled data, which may not always be available, especially in medical imaging tasks. Transfer learning mitigates this challenge by using pre-trained models that have already learned generic features and achieves high accuracy even with a relatively small dataset, such as the one used in this study.
- Fine-tuning: Transfer learning allows for fine-tuning pre-trained models on a specific task. In the proposed framework, models like DenseNet201 were fine-tuned using oral cancer histopathology slide images. Fine-tuning involves updating the model’s weights and parameters to adapt to the specific characteristics of the new dataset.
- Speed and efficiency: Transfer learning reduces the training time and computational resources compared to training a deep model from scratch.

The efficacy of deep learning (DL) models is profoundly contingent upon the volume of accessible data and the strategic selection of hyperparameters. The hyperparameter configuration substantially impacts a convolutional neural network’s performance, and suboptimal selections can detrimentally affect applications [28]. Instead of adopting a random approach to determining hyperparameter values, an optimization procedure is implemented to fine-tune these parameters [28] meticulously. Such optimization ensures that hyperparameters are modulated proficiently, enhancing the application’s performance.

As discussed earlier, optimization methods are vital in various fields, including engineering, mathematics, medicine, and the military. They are crucial in enhancing efficiency and effectiveness by finding optimal solutions to recurrent problems across different domains [29]. These methods are particularly valuable when applied to real-world scenarios, where finding the best solution can have significant practical implications. In medical applications, preprocessing and optimization techniques have gained increasing attention from healthcare professionals; specifically, the automated classification of diseases, such as the early detection of oral cancer. However, diagnosing these conditions accurately and efficiently can be challenging. In the preprocessing and optimization stages of building a deep learning system for tasks such as oral cancer classification, several challenges are commonly encountered:

- Data preprocessing challenges:
  - Data quality: Histopathology slide images may have varying qualities due to image resolution, staining variations, and artifacts.
  - Data augmentation: Augmenting the dataset by creating variations of the original images is essential for training deep learning models effectively. However, determining which augmentation techniques to apply and their parameters can be challenging.
- Hyperparameter optimization challenges:
  - High-dimensional hyperparameter space: Deep learning models have numerous hyperparameters, including learning rates, batch sizes, dropout rates, activation functions, and more. The hyperparameter space is high-dimensional, making manual tuning impractical.
  - Computational resources: Conducting an exhaustive search of the hyperparameter space can be computationally expensive and time-consuming, especially when dealing with multiple models and configurations.
  - Overfitting: Optimizing hyperparameters can lead to overfitting, where the model performs exceptionally well on the training data but fails to generalize to new, unseen data.
- Model selection challenges:
  - Model complexity: Choosing the appropriate deep learning architecture for the task is crucial. Models vary in complexity, and selecting one that balances performance and computational cost is challenging.
  - Transfer learning: Another challenge is deciding whether to use transfer learning and selecting the most suitable pre-trained model. Not all pre-trained models are equally effective for every task.
- Optimizer selection challenges:
  - Optimizer diversity: There is a wide variety of optimization algorithms available for deep learning, including gradient-based methods, evolutionary algorithms, and metaheuristic optimizers.
  - Optimizer hyperparameters: Optimizers have hyperparameters that need tuning, such as learning rates and momentum. Determining the optimal values for these hyperparameters is challenging.
- Evaluation metrics: Choosing appropriate evaluation metrics to assess the performance of the models is essential. In medical applications such as oral cancer classification, metrics like accuracy, sensitivity, specificity, and area under the ROC curve (AUC) are commonly used, but selecting the most relevant ones is challenging.

This is where optimization techniques come into play. By automating the classification of lesions based on medical imaging data, optimization algorithms can assist healthcare providers in making timely and accurate diagnoses. The optimization process [29] is iterative and involves an extensive search for the best solution among various trial alternatives. Optimization techniques can be broadly categorized into deterministic and stochastic algorithms [29,30]. Deterministic methods find globally optimal solutions quickly but may suffer from performance degradation as the problem size increases. These methods are complex and specialized [31] and struggle with NP-hard multidimensional problems.

On the other hand, stochastic optimizers, types of stochastic algorithms, use randomness to explore solutions broadly, although they do not guarantee optimal results [31]. Heuristic approaches, like evolutionary algorithms, memetic algorithms, and greedy strategies, fall under this category, providing efficient, near-optimal solutions at a lower cost [31]. However, many of these heuristics are problem-specific.

Metaheuristic algorithms, a class of stochastic algorithms inspired by biological systems, excel in solving nonlinear, multidimensional optimization problems [32]. They offer accurate and robust solutions, and their problem-independent nature makes them adaptable to various design challenges. Metaheuristics tackle complex, intractable problems at a higher level of abstraction [31] without depending on preconditions like differentiability or continuity.

Metaheuristics have several advantages: they do not require gradient information, can be adjusted dynamically, and are flexible due to their black-box design. These procedures start with trial-and-error approaches, evaluate potential solutions based on algorithm-specific equations, and continue until a predetermined stopping criterion is met [31]. As a result, different optimization techniques can yield solutions with varying levels of improvement.

Metaheuristic optimization involves a two-stage approach to finding optimal solutions: diversification (exploration) and intensification (exploitation). Diversification aims to maintain a global search by reducing the risk of getting stuck in local minima through randomizing the search. Intensification evaluates promising solutions near the population memory, akin to a targeted local search. Balancing these stages is crucial for effective metaheuristic optimization.

The no free lunch (NFL) theorem states that all nonsampling algorithms are roughly as effective as one another in solving practically any optimization issue. The theory holds that any given black-box search or optimization algorithm will produce the same results across various target functions within a constrained search space [32]. However, the issue with algorithms is that they cannot effectively tackle every real-world situation. In the end, the NFL theorem has the potential to derail the efforts of the researcher who seeks to create a super-algorithm that solves all issues faster than a random algorithm.

Nature-inspired metaheuristic algorithms simulate biological or physical phenomena to solve optimization problems. The algorithms can be broken down into five classes: physics-based, nature-based, human-based, swarm-based, and animal-based. Researchers have shown that most metaheuristic algorithms are inspired by the strategies employed by predators and prey in the wild.

Three of the most used metaheuristic algorithm types are based on evolution, physics, and swarms [30]. The swarm algorithm is a model that may be used to simulate the social behavior of a population. Various optimization algorithms based on swarms have been developed since the early 1990s, including particle swarm optimization (PSO) and ant colony optimization (ACO). Swarm intelligence algorithms include, but are not limited to, artificial bee colony algorithms, firefly optimization algorithms, grey wolf optimization algorithms, sparrow optimization methods, and whale optimization algorithms.

The artificial Gorilla Troops Optimizer (GTO) is a recently released metaheuristic optimization method inspired by gorillas’ natural behavior. Abdollahzadeh et al. [33,34] developed the GTO in 2021. The technique simulates gorillas’ social behavior and movements in the wild. Gorillas live in family units known as “troops”, which typically include a dominant male known as a “silverback”, as well as many females and their young [32]. The GTO stands out due to its unique inspiration from the social behavior of gorilla troops. The GTO introduces a novel optimization approach by simulating the dynamics of gorilla family units led by dominant silverbacks and considering the interactions between different members. This algorithm leverages the division of roles within gorilla troops, mimicking these groups’ cooperation and decision-making processes. GTO offers a fresh perspective on optimization, potentially enhancing its performance in solving complex problems. Its adaptability and emulation of nature’s strategies make it valuable to the optimization toolkit.

The Aquila Optimizer (AO) is an optimization algorithm that takes its cues from the natural world and is inspired by the activity of hunting. As one of the most well-known raptors, the aquila is a common sight. Aquilas can capture a wide range of ground-dwelling prey due to their swiftness, agility, sturdy feet, and large sharpened talons. Aquila employs four main hunting methods, each with advantages and disadvantages; most can switch between them quickly and intelligently depending on the circumstances [35,36,37].

The AO algorithm emulates aquilas’ actions at each hunting stage to show how the bird operates under pressure. An overview of the AO algorithm’s four main steps reveals that it involves high soaring with a vertical stoop to select the search area, contour flight with short glide attacks to locate within divergent search areas, low soaring with a slow descent to exploit within convergent search areas, and walk-and-grab attacks to swoop in and grab targets within convergent search areas. AO has two phases of updating the current individuals: exploration and exploitation, as do other metaheuristic techniques. Furthermore, the AO algorithm can employ alternative behaviors to transition from the exploration phase to the exploitation phase based on this condition: $if(t\leq \frac{2}{3}T)$ the exploration steps will be enabled; otherwise, the exploitation steps will take place [35,38]. The exploration phase occurs when $if(t\leq \frac{2}{3}T)$, and it contains two methods; the first one is expanded exploration while the second is narrowed exploration.

The AO draws inspiration from the hunting tactics of the aquila, a raptor known for its agility and efficiency in capturing prey. The AO’s ability to replicate these hunting strategies provides a versatile optimization approach. By dynamically transitioning between these methods based on specific conditions, AO introduces an element of adaptability not commonly seen in other metaheuristic algorithms. This adaptability enables AO to optimize solutions effectively across various problems and complexities. The Aquila Optimizer (AO) contributes to the system’s accuracy by efficiently tuning and optimizing the various hyperparameters of the deep learning models used in the oral cancer classification system. The AO can enhance the accuracy as follows:

- Hyperparameter optimization: DL models have numerous hyperparameters that significantly impact performance. These hyperparameters include learning rates, batch sizes, dropout rates, activation functions, and more. Manually tuning these hyperparameters can be time consuming and may not yield the best results. The AO automates this process by intelligently searching the hyperparameter space to find the optimal configuration for each model. This fine-tuning leads to improved accuracy.
- Loss function selection: AO recommends using specific loss functions for different models. The choice of loss function is crucial in training deep learning models. Different loss functions are suitable for different tasks and datasets.
- Model selection: The AO might also assist in selecting the most appropriate pre-trained convolutional neural network (CNN) model for the task. Different CNN architectures have varying levels of complexity and are better suited for specific types of data.
- Robustness to data variability: Like many medical datasets, oral cancer datasets can be highly variable due to differences in patient populations and image quality. The AO helps make the models robust to this variability by finding hyperparameter configurations that work well across different subsets of the data.
- Optimal data augmentation: The AO can also guide the decision to use data augmentation techniques. Data augmentation involves creating variations of the original data to improve the model’s ability to generalize to unseen examples. The AO can determine whether data augmentation would benefit each model, further enhancing accuracy.

These optimization techniques, rooted in the principles of artificial intelligence, stochastic algorithms, and nature-inspired metaheuristics, lay the groundwork for the application of automated classification of oral cancer. By harnessing the power of deep learning and optimization, we aim to enhance the accuracy and efficiency of diagnosing oral cancer, ultimately improving patient outcomes and advancing medical diagnostics.

## 3. Related Studies

The advent of machine learning achieved tremendous changes in medical imaging analysis by developing robust approaches to tackle medical image classification issues and providing computer-aided diagnosis systems that reduce observer-specific variability [16]. Furthermore, CNNs demonstrate the possibility of automating the classification of various cancerous tumors. Herein, two major techniques can be devolved; the first is based on manual feature extraction according to pathologists’ knowledge to ascertain grading. The second technique is based on deep learning without manual feature engineering. As a result, image classification has benefited greatly from deep learning by developing an architecture that meets the classification challenges and increases the predictable outcomes.

OSCC can be detected early, which helps reduce cancer-related mortality and morbidity [39]. Unfortunately, oral cancer is identified at an advanced stage in the majority of instances. The histopathological examination is the standard procedure for diagnosing OSCC; however, tumor heterogeneity constitutes a major challenge [11]. The increasing application of digitalization in histopathology motivates extensive research on developing accurate deep-learning-based decision support systems that can help in OSCC prognosis and management. Obtaining reliable diagnostic and prognostic information for OSCC could greatly assist pathologists in making informed judgments that assure effective healthcare screening support, early detection, and treatment. This section reviews the up-to-date state-of-the-art studies that have applied deep learning in OSCC.

Aubreville et al. [40] proposed a deep artificial neural network (DNN) approach for the automatic binary classification of OSCC. The approach was based on 7894 oral cavity CLE images from OSCC patients. The proposed approach first preprocessed images by grouping images into patches and scaling them to reduce processing complexity and noise. Data augmentation (DA) was then performed to enrich the data. Finally, classification approaches were deployed using SVM and RF. The approach achieved image recognition with an accuracy of 88.3%. However, this approach needs further enhancement, especially in accuracy and adapting it for more complex diagnosis tasks. Ariji et al. [41] investigated a deep learning CT image classifier to diagnose oral cancer lymph node metastasis. They used CT images of 441 histological nodes from 45 patients with OSCC. Their approach involved segmentation by two experienced radiologists, augmentation, training with a five-fold cross-validation procedure, validation, and testing using the AlexNet architecture. Using the DCNN classification algorithm, they achieved an accuracy of 78.2%.

Jeyaraj and Nadar [14] presented a partitioned DCNN (PDCNN) model for detecting cancerous lesions in hyperspectral images. The PDCNN model was developed to classify RoI in the multidimensional hyperspectral image. The model involves classification, segmentation, labeling, feature extraction, and deep learning algorithms. They used a dataset from three repositories consisting of 2200 images. The proposed partitioned DCNN outperformed the conventional SVM classification technique and achieved a classification accuracy of 94.5% using the selected bagging and boosting method that selects the final feature based on weighted votes. Motivated by building a lightweight oral lesions classifier, Jubair et al. [39] introduced a transfer-learning-based DCNN. They used the EfficientNet-B0 transfer model for binary classification. For training and testing, 716 clinical images for tongue lesions were used. The experimental analysis reported an accuracy of 90%. The limitation of this study was relying on a small dataset that contained tongue lesions only. An OSCC classification using the MobileNet CNN for FCM scanner images was demonstrated in [42]. Tissue samples from twenty patients were collected and identified based on the location and histological grading, and then an ex vivo FCM investigation proceeded. After that, tissue annotation and feature extraction were performed. The model achieved a specificity of 96%. The main drawback of this work was using small sample sizes.

An automated oral lesions binary classification deep learning technique was proposed using the combined ResNet50 and VGG16 models in [43]. The model was trained with 332 oral lesion digital images. These images were processed by discrete wavelet transform and adaptive histogram equalization. The ensemble model, capable of effectively extracting all useful features, achieved an accuracy of 96.2%. Jelena et al. [11] introduced a CNN-based dual-stage OSCC diagnostic system. The system performed OSCC multiclass classification and segmentation. In the first stage, three automated classes of tumor grading were performed using Xception and SWT. In the second stage, microenvironment cell segmentation was used to discover new features. The workflow started with image acquisition, preprocessing, and augmentation. Then, decomposition, semantic segmentation, and classification. Finally, the best-performing configuration was deployed. The ensemble resulted in a classification accuracy of 94.1%. In [8], the authors aimed to construct a simple and reliable ANN model for classifying oral cancer based on risk factors, systematic medical problems, and clinical pathology aspects. A dataset consisting of 73 patients with 29 variables/cases was used. The analysis demonstrated a classification accuracy of 78.95%. The proposed model’s biggest flaw was using a too-small database.

Panigrahi et al. [16] studied applying a deep learning architecture called a capsule network (CN) for OSCC diagnosis using histopathological images. For classification, the CN was based on a dynamic routing algorithm. Five distinct processes make up the proposed method: preprocessing, segmentation, image, augmentation, data partitioning, and binary classification. This method achieved 97.35% accuracy using a WSI of 150 images. In [1], the authors used a CNN to classify and segment OSCC from H&E-stained histological WSIs. The first stage was preprocessing to remove background and scanning artifacts and segment RoI to extract and quantify features. A new dataset involving two types of WSI containing 85,621 image patches for OSCC tissue samples and breast cancer metastases was introduced. The method achieved an accuracy of 97.6%, and the preprocessing stage needed further optimization. In [44], Maurya et al. introduced a TL-based classification approach for multiclass OSCCs based on microscopic imaging. The framework extracted features from the three ensembles of DCNN models that applied various optimization methods. The framework was trained and tested on five large public datasets. A classification accuracy of 99.28% was achieved for the ensemble-based approach.

A segmentation and classification approach for detecting oral dysplasia lesions (ODLs) was introduced in [44]. The approach involved four stages: segmentation using an R-CNN, post-processing using morphological operations, feature extraction, and classification using a polynomial classifier. In this method, 66 images of the tongues of mice were histologically divided into 296 sections. The segmentation and classification accuracy ranged from 88.92% to 90.35%. In addition, preprocessing techniques could reduce the impact of pigmentation excesses or deficiencies. A multiclass OSCC grading was proposed in [45], using multiple DCNN architectures. A five-stage architecture was proposed based on TL with four pre-trained models and a CNN model. The workflow started with the acquisition, labeling, augmentation, segmentation, and classification. The proposed CNN model obtained an accuracy of 97.5% with 156 histopathological WSI datasets, and a large-scale training dataset was needed. Figueroa et al. [22] developed a deep learning training approach to achieve understandability. This study was focused on utilizing gradient-weighted class activation mapping and a dual training process with augmentation for optimized classification and segmentation. First, they collected the dataset and performed data cleaning, labeling, and annotation. Two-stage training was then performed; in the first stage TL was used on VGG19 as well as data augmentation, and in the second stage the GAIN training architecture was deployed. Although a classification accuracy of 86.38% was achieved, further enhancement is required.

The application of DS approaches has already shown that they have the potential to revolutionize medical care in the fields of imaging, surgery, and laboratory medicine [46]. Numerous deep learning architectures have been proposed concerning OSCC automatic detection; however, they have several issues and challenges. For example, most of the proposed approaches lack highly complete datasets, have high system operation costs, suffer from limited accuracy, and fail optimization. Accordingly, further studies are needed to develop enhanced and optimized architectures to be integrated into clinical practices. Future research in OSCC detection techniques could focus on the following:

- Large-scale datasets: Collecting and annotating large-scale datasets with diverse OSCC cases to train more robust deep learning models.
- Reducing operational costs: Exploring cost-effective data acquisition methods and model deployment in clinical settings.
- Improving accuracy: Investigating advanced network architectures, ensemble methods, and hybrid approaches to enhance classification accuracy.
- Clinical integration: Achieve seamless integration of deep learning models into clinical workflows, ensuring practical utility.
- Addressing dataset bias: Addressing potential biases in training data that may affect model generalization.

Further research and improvements in OSCC detection techniques promise to revolutionize oral cancer diagnosis and management, ultimately improving patient outcomes. This comprehensive review of related studies informs the approach taken in this manuscript, where the metaheuristic optimization algorithms GTO and AO were proposed to enhance the accuracy and efficiency of OSCC detection from histopathological images. The subsequent sections will detail the methodology and experimental setup based on the insights gained from the reviewed studies.

## 4. Methodology

**Figure 4:**
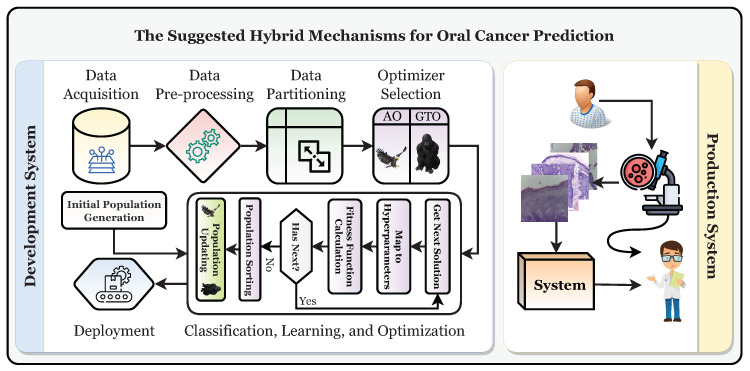
The suggested development and production framework.

This study establishes a framework for the automatic and trustworthy classification of oral cancer based on histopathological microscopic examination slides with the aid of a CNN, transfer learning, and two metaheuristic optimizers (i.e., AO and GTO) for optimizing the parameters and hyperparameters. Figure 4 depicts the proposed development and production framework.

Figure 4 shows that the patient will have a biopsy performed in the oral examination to take the histopathological slides. The classification of the slide will be performed using the classifier, and it should be diagnosed as “normal” or “OSCC” (i.e., oral squamous cell carcinoma). After that, the specialist will decide based on their and the system’s decisions. To reach the production system, the development system must be run first to obtain the final optimized and state-of-the-art classifier. It can be broken down into six distinct stages: the data acquisition phase, the preprocessing phase, the partitioning phase, the classification phase, the learning phase, and the optimization phase, in addition to the deployment phase.

### 4.1. Phase 1: Data Acquisition

**Figure 5:**
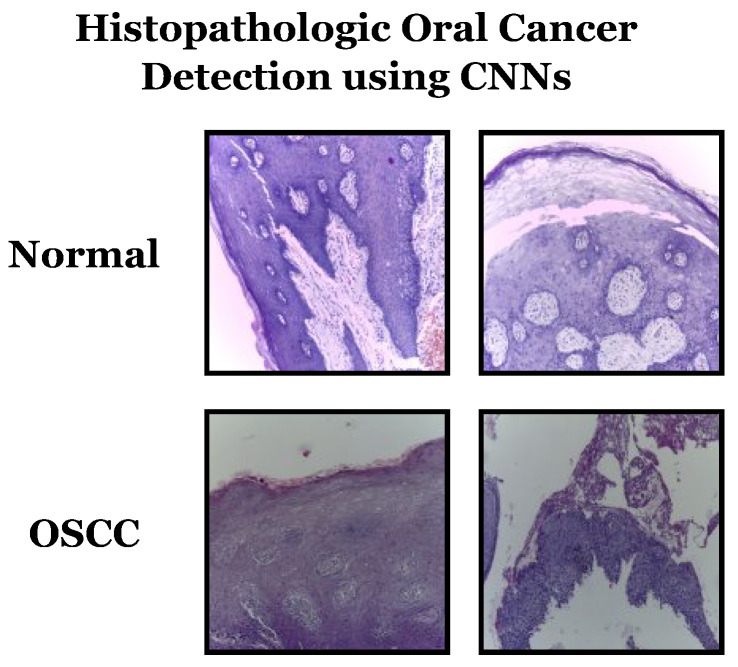
Samples from the used dataset.

Datasets can be sourced from various places, including offline sources like hospitals and online sources such as repositories. This study utilizes the latter method and the “Histopathologic Oral Cancer Detection using CNNs” dataset, available on Kaggle. Representative samples from the dataset can be seen in Figure 5. Some characteristics of the dataset include:

- Dataset name: Histopathologic Oral Cancer Detection using CNNs.
- Categories: The dataset consists of two main categories:
  - “Normal”: This category contains histopathology slide images representing normal oral tissue.
  - “OSCC” (oral squamous cell carcinoma): This category contains histopathology slide images representing oral tissue with squamous cell carcinoma, a type of oral cancer.
- Dataset size: The dataset contains a total of 5192 histopathology slide images, with a split between the two categories as follows:
  - “Normal”: 2494 images.
  - “OSCC”: 2698 images.
- Data balance: The dataset appears reasonably balanced, with a relatively similar number of images in both the “normal” and “OSCC” categories. Balanced datasets are essential for training machine learning models effectively.
- Image content: Each image in the dataset represents a histopathology slide capturing microscopic tissue details. These images are used to diagnose and classify normal and cancerous oral tissue.
- Purpose: The dataset is specifically designed for oral cancer detection using convolutional neural networks (CNNs) and serves as the primary data source for evaluating the proposed framework in the study.

### 4.2. Phase 2: Data Preprocessing

During the second phase, three distinct preprocessing techniques will be applied to the datasets. These techniques involve resizing, adjusting dimension scales, and achieving balance.

#### 4.2.1. Process 2.1: Data Resizing

The target collection’s images come in different sizes, so it is necessary to resize them to ensure uniform dimensions. For this study, a size of (128,128,3) was chosen, and the resizing was performed in RGB color space using the bicubic interpolation method.

#### 4.2.2. Process 2.2: Data Scaling

This study employs four scaling methods: normalization, standardization, min–max scaling, and max-abs scaling. These methods are referred to as Equations (1), (2), (3), and (4), respectively.

(1) $$X_{output}=\frac{X}{\max\left(X\right)}$$

(2) $$X_{output}=\frac{X-\mu }{\sigma }$$

(3) $$X_{output}=\frac{X-\min\left(X\right)}{\max\left(X\right)-\min\left(X\right)}$$

(4) $$X_{output}=\frac{X}{\left|\max\left(X\right)\right|}$$

where *X* is the input image, $X_{output}$ is the scaled image, μ is the image mean, σ is the image standard deviation.

#### 4.2.3. Process 2.3: Dataset Balancing

The dataset used in this study is skewed since there is an uneven distribution of images across categories. Data augmentation is used to expand and standardize the number of images in each category before beginning the training process, which helps with this problem. As a result of this equalization, the dataset comprises 5396 images, with each class having 2698 images. This research employs various methods such as rotation, translation, shearing, zoom, flip, and brightness augmentation, as described in [47]. Table 1 displays the augmentation strategies and configurations used to balance the dataset.

### 4.3. Phase 3: Data Partitioning

The dataset is split into three parts: training, testing, and validation, with a split ratio of 0.85. The first step is to create a training set and a validation set from the complete dataset with 85% and 15% of the records, respectively. Then, the training set is further divided into two parts, with 85% of the records going to training and 15% going to validation. As a result, the record counts for training are 72.25%, for validation 12.75%, and for testing 15%.

### 4.4. Phase 4: Classification, Learning, and Optimization Phase

The learning phase starts once the datasets have undergone preprocessing. During this phase, the AO and GTO metaheuristic optimizers optimize transfer learning hyperparameters such as the batch size. This method aims to find the best hyperparameter combinations that produce the best results for each transfer learning model being used. This approach is better than a random search or grid search. Three different processes are involved, summarized in the algorithm referred to in Algorithm 1. In the optimization realm, particularly in the intricate domain of hyperparameter tuning for deep learning models, the Gorilla Troops Optimizer (GTO) and Aquila Optimizer (AO) emerge as potent algorithms, each embodying distinctive characteristics and advantages. The AO is characterized by its adept exploration capabilities, facilitated by strategic population initialization and update mechanisms, ensuring a robust and comprehensive search space exploration. Conversely, the GTO distinguishes itself through a troop-based strategy, effectively exploiting promising regions of the search space and ensuring a meticulous convergence towards optimal solutions by avoiding premature convergence. Both algorithms exhibit a commendable balance between exploration and exploitation, ensuring that the search does not stagnate in local optima and continues exploring diverse parameter space regions. Moreover, their adaptability and robustness are evidenced by their applicability and consistent performance across many optimization problems and various convolutional neural network models. Thus, the GTO and AO stand out as versatile and efficient metaheuristic optimization algorithms, offering reliable and enhanced performance in optimizing hyperparameters, thereby contributing significantly to the advancement of automated and accurate oral cancer detection through deep learning. The latter procedures can be performed as many times as $T_{max}$, but the first procedure is only performed once.
**Algorithm 1:** A summary of the overall process of hyperparameter optimization
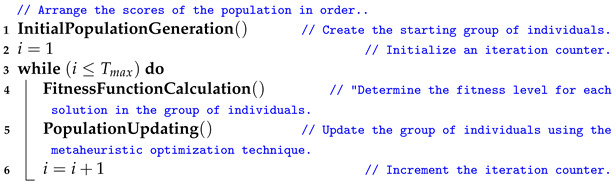


#### 4.4.1. Initial Population Creation

A group of $N_{max}$ solutions is randomly created at the beginning of the learning phase. Each solution is represented as a vector with 1×D dimensions, with all elements falling within the range of [0, 1]. The random generation of the population is shown in Equation (5).

(5) $$X=rand\times \left(UB-LB\right)+LB$$

where *X* denotes the whole population, LB is the lower boundaries for each solution, UB is the upper boundaries for each solution, and rand is a random value [48].

Each component of the solution represents a certain hyperparameter. The relationship between the solution index and the corresponding hyperparameter is shown in Table 2. The table shows that if data augmentation is used, *D* will equal 15; if not, *D* will equal 7.

#### 4.4.2. Fitness Function Calculation

In this step, the performance of each solution is evaluated by calculating its fitness function score. The solution is transformed into the corresponding hyperparameters, as outlined in Table 2. To understand how this conversion works, let us take the batch size (the second element) as an example. We must first determine the range of acceptable batch sizes to find the appropriate batch size. In this study, the range used is “4→48(step=4)”, resulting in 12 possible options. The mapping process can be calculated using Equation (6) to determine the correct option.

(6) $$\mathrm{Span}\;\mathrm{Index}=\left\lceil \mathrm{Length}\left(\mathrm{ranges}[index]\times \mathrm{solution}[index]\right)\right\rceil$$

The conversion of a random numeric value to its corresponding hyperparameter index is described in Equation (6). When there are twelve possible random numbers, one of those numbers is 0.75, the index is nine, and the batch size is 36. It is important to note that the range of values for each hyperparameter can be found in Table 3.

The solution is used to assemble the pre-trained TL model for the target using the translated hyperparameters. The current study utilizes the pre-trained transfer learning models Xception, VGG16, VGG19, MobileNet, MobileNetV2, MobileNetV3Small, MobileNetV3Large, NASNetMobile, and DenseNet201, all of which have “ImageNet” pre-trained weights. In the current study, the pre-trained TL model will initiate training on the divided datasets for an iteration count of five.

Its efficacy can be assessed only through testing the pre-trained TL model on the full dataset. The effectiveness of a model can be evaluated in several ways, including by looking at measures like accuracy, the area under the curve (AUC), and specificity.

This study uses various performance indicators, including accuracy, precision, specificity, recall (i.e., sensitivity), F1 score, AUC, IoU, the Dice coefficient, cosine similarity, Youden Index, and NPV. These performance metrics are defined in Equations (7)–(14).

(7) $$\mathrm{Accuracy}=\frac{TP+TN}{TP+TN+FP+FN}$$

(8) $$\mathrm{Precision}=\frac{TP}{TP+FP}$$

(9) $$\mathrm{Specificity}=\frac{TN}{TN+FP}$$

(10) $$\mathrm{Recall}=\mathrm{Sensitivity}=\frac{TP}{TP+FN}$$

(11) $$\mathrm{Dice}\;\mathrm{Coef}.=\frac{2\times TP}{2\times TP+FP+FN}$$

(12) $$F1\text{-}\mathrm{score}=\frac{2\times \mathrm{Precision}\times \mathrm{Recall}}{\mathrm{Precision}+\mathrm{Recall}}$$

(13) YoudenIndex=Specificity+Sensitivity−100%

(14) $$\mathrm{NPV}=\frac{TN}{TN+FN}$$

#### 4.4.3. Population Updating

The solutions are ranked in descending order based on their fitness scores (placing the best solution first and the worst solution last). This is important in determining the best and worst solutions ($X_{best}^{t}$ and $X_{worst}^{t}$) if they are needed in the update process.

The current study uses two metaheuristic optimization algorithms for comparison. The first is AO, which operates through four stages: expanded exploration, narrowed exploration, expanded exploitation, and narrowed exploitation. Equation (15) represents the process of expanded exploration. Equation (16) represents the process of narrowed exploration. Equation (17) represents the process of expanded exploitation. Equation (18) represents the process of narrowed exploitation.

(15) $$X_{AO1}\left(t+1\right)=X_{best}\left(t\right)\times \left(1-\frac{t}{T_{max}}\right)+\left(X_{M}\left(t\right)-X_{best}\left(t\right)\times rand\right)$$

(16) $$X_{AO2}\left(t+1\right)=X_{best}\left(t\right)\times \mathrm{Levy}\left(D\right)+X_{R}\left(t\right)+\left(y-x\right)\times rand$$

(17) $$X_{AO3}\left(t+1\right)=\left(X_{best}\left(t\right)-X_{M}\left(t\right)\right)\times \alpha -rand+\left((UB-LB)\times LB\right)\times \delta$$

(18) $$X_{AO4}\left(t+1\right)=QF\times X_{best}\left(t\right)-\left(G_{1}\times X\left(t\right)\times rand\right)-G_{2}\times \mathrm{Levy}\left(D\right)+rand\times G_{1}$$

The equation used in the study describes the operation of the AO metaheuristic optimizer. The variables in the equation include $X^{t}$, which is the solution at iteration *t*; rand, which is a random number within the range of [0,1]; $X_{M}\left(t\right)$ is the mean of the locations; Levy(D), which is the Levy flight distribution function; $X_{R}\left(t\right)$, a random solution; *y* and *x* represent a spiral shape in the search; α and δ are exploitation adjustment parameters; QF, a quality function used to balance the AO search strategies; $G_{1}$, representing various motions of the AO used to track the prey during the escape; and $G_{2}$ which is a decreasing value from 2 to 0 [49].

The second metaheuristic optimizer used in the study is GTO, which operates through (1) three exploration strategies, (2) an exploitation mechanism, and (3) a mechanism for competition among adult females. The expanded exploration process is represented by Equation (19), the exploitation mechanism by Equation (20), and the competition among adult females by Equation (21).

(19) $$X_{GTO1}\left(t+1\right)=\left\{\begin{array}{cc} LB+\left(UB-LB\right)\times r_{1}, & \mathrm{if}\;(rand<p)\\ L\times H+\left(r_{2}-C\right)\times X_{r}\left(t\right), & \mathrm{if}\;(rand\geq 0.5)\\ X\left(i\right)-L\times \left(L\times \left(X\left(t\right)-X_{r}\left(t\right)\right)+r_{3}\times \left(X\left(t\right)-X_{r}\left(t\right)\right)\right), & \mathrm{Otherwise} \end{array}\right.$$

(20) $$X_{GTO2}\left(t+1\right)=L\times M\times \left(X\left(t\right)-X_{silverback}\right)+X\left(t\right)$$

(21) $$X_{GTO3}\left(t+1\right)=X_{silverback}-\left(X_{silverback}\times Q-X\left(t\right)\times Q\right)\times A$$

The variables in the equations include $r_{1}$, $r_{2}$, and $r_{3}$, which are random values; $X_{r}\left(t\right)$, representing a random solution; $X_{silverback}$, which is the position vector of the best solution (i.e., silverback gorilla); *Q*, which simulates the impact force; and *A*, a coefficient vector that determines the level of violence in conflicts.

### 4.5. The Suggested Framework Pseudocode

The steps are repeated $T_{max}$ times. Once the iterations have finished, the optimal combination can be applied to other systems or analyses. Algorithm 2 summarizes the proposed method for optimizing parameters and hyperparameters.

The time complexity of Algorithm 2 depends on several factors, including the number of iterations ($T_{max}$), the number of solutions considered ($N_{max}$), the complexity of the fitness score calculation, and the complexity of the solution update step using the metaheuristic optimization technique. For the number of iterations ($T_{max}$), the outer loop runs for a maximum of $T_{max}$ iterations. If $T_{max}$ is a fixed constant, this part contributes $O(T_{max})$ to the time complexity. For the number of solutions ($N_{max}$), the inner loop calculates fitness scores for each of the $N_{max}$ solutions. Therefore, it has a time complexity of $O(N_{max})$.

The time complexity of calculating the fitness score for a single solution depends on the complexity of training and evaluating the TL model. This part depends on the model architecture, dataset size, and the number of epochs during training. Let us denote this as O(fitness). The solution update step using the metaheuristic optimization technique may involve modifying the hyperparameters of the TL model. The time complexity of this step depends on the optimization algorithm and how it explores the hyperparameter space, denoted as O(update). Thus, the dominant factors in the time complexity of Algorithm 2 will be $T_{max}$, $N_{max}$, and the complexities of the fitness calculation and solution updates.
**Algorithm 2:** The proposed framework pesudocode
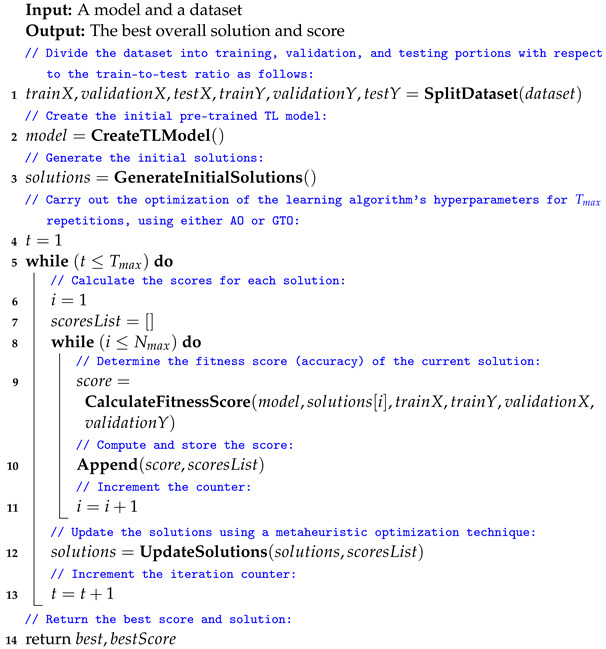


## 5. Experimental Results

Table 3 summarizes the common configurations of the experiments performed in this study. In the experimental process described in the study, pre-trained transfer learning (TL) models are applied as follows:

- Selection of pre-trained TL models: The study mentions the use of nine pre-trained convolutional neural network (CNN) models: NASNetMobile, Xception, VGG16, VGG19, DenseNet201, MobileNetV2, MobileNetV3Small, MobileNet, and MobileNetV3Large. These models are chosen based on their effectiveness in image classification tasks and availability with pre-trained weights from the ImageNet dataset.
- Transfer learning: The pre-trained TL models have already been trained on a large dataset (ImageNet) for general image recognition. The study employs transfer learning by taking these pre-trained models as a starting point.
- Fine-tuning: After selecting a pre-trained TL model, the study fine-tunes it for classifying oral cancer using histopathology slide images. Fine-tuning involves modifying the architecture and updating the model’s parameters to better fit the characteristics of the oral cancer dataset. This process helps the model learn relevant features from the medical images.
- Hyperparameter optimization: The optimization process determines the best hyperparameters for each pre-trained TL model. This includes parameters related to the model architecture, learning rate, batch size, and other training-related settings. The Gorilla Troops Optimizer (GTO) and Aquila Optimizer (AO) are used to search for optimal configurations efficiently.
- Evaluation: After fine-tuning and hyperparameter optimization, the performance of each pre-trained TL model is evaluated using various metrics such as accuracy, AUC, and specificity. These metrics help to assess how well the models can classify oral cancer from histopathology slide images.

### 5.1. The Aquila Optimizer (AO) Experiments

For a summary of the top configurations and results for the dataset and AO metaheuristic optimizer, see Table 4 and Table 5, respectively. Seven models favor the KL divergence loss function, as seen in the above-mentioned tables. The SGD Nesterov and AdaMax parameter optimizers are recommended by three models each. The max-abs and standardization scaling techniques are also recommended by three models each. Finally, applying data augmentation is recommended by seven models.

Table 5 shows that the average accuracy is 99.25%, the average F1 score is 99.25%, the average precision is 99.25%, the average recall is 99.25%, the average specificity is 99.25%, the average AUC is 99.77%, the average sensitivity is 99.25%, the average IoU is 98.97%, the average Dice coefficient is 99.15%, the average cosine similarity is 99.30%, the average Youden index is 98.50%, and the average NPV is 99.25%.

### 5.2. The Artificial Gorilla Troops Optimizer (GTO) Experiments

Table 6 summarizes the best configurations and Table 7 summarizes the best results related to the used dataset and the GTO metaheuristic optimizer.

Table 6 shows that the Poisson loss function is recommended by four models. The SGD Nesterov and AdaMax parameter optimizers are recommended by three models each. The standardization scaling technique is recommended by six models. Applying data augmentation is recommended by seven models.

Table 7 shows that the average accuracy is 97.27%, the average F1 score is 97.27%, the average precision is 97.27%, the average recall is 97.27%, the average specificity is 97.27%, the average AUC is 99.23%, the average sensitivity is 97.27%, the average IoU is 96.36%, the average Dice coefficient is 97.03%, the average cosine similarity is 97.65%, the average Youden index is 94.55%, and the average NPV is 97.27%.

### 5.3. AO vs. GTO Analysis

**Figure 6:**
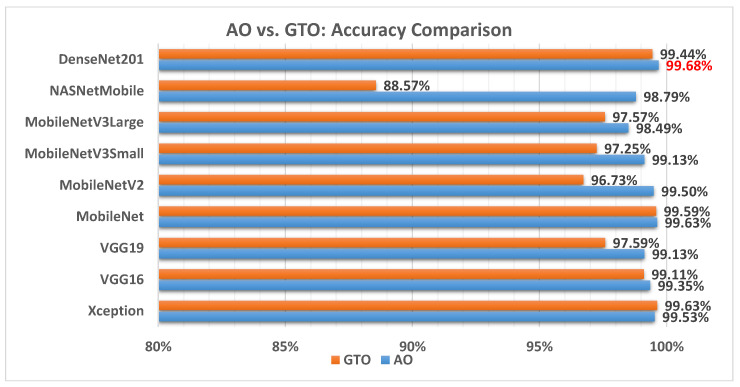
A comparison between the AO and GTO concerning the accuracy.

Figure 6 illustrates a comparison between the performance of the AO and GTO with respect to accuracy. From the accuracy point of view, the AO is better than the GTO in all models except for the Xception model. The best model is DenseNet201.

**Figure 7:**
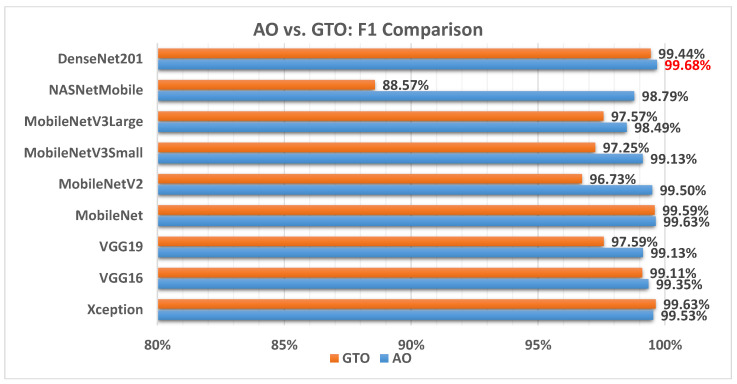
A comparison between the AO and GTO concerning the F1 score.

Figure 7 illustrates a comparison between the performance of AO and GTO with respect to the F1 score. From the F1 score point of view, the AO is better than the GTO in all models except for the Xception model. The best model is DenseNet201.

**Figure 8:**
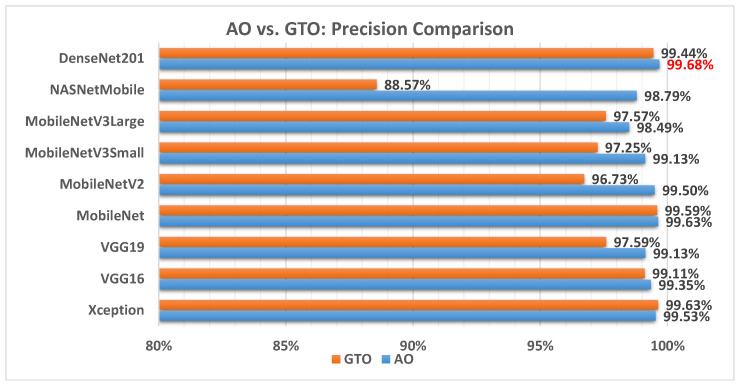
A comparison between the AO and GTO concerning the precision.

Figure 8 illustrates a comparison between the performance of the AO and GTO with respect to precision. From the precision point of view, the AO is better than the GTO in all models except for the Xception model. The best model is DenseNet201.

**Figure 9:**
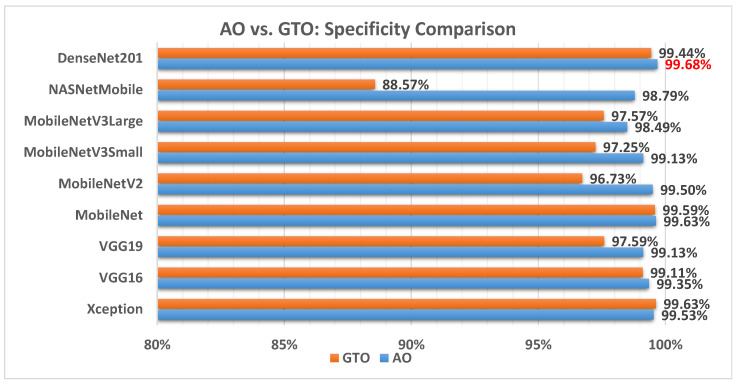
A comparison between the AO and GTO concerning the specificity.

Figure 9 illustrates a comparison between the performance of the AO and GTO with respect to specificity. From the specificity point of view, the AO is better than the GTO in all models except for the Xception model. The best model is DenseNet201.

**Figure 10:**
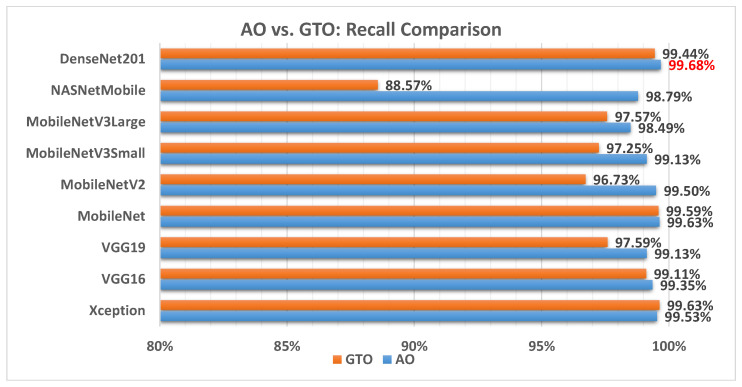
A comparison between the AO and GTO concerning the recall.

Figure 10 illustrates a comparison between the performance of the AO and GTO with respect to recall. From the recall point of view, the AO is better than the GTO in all models except for the Xception model. The best model is DenseNet201.

**Figure 11:**
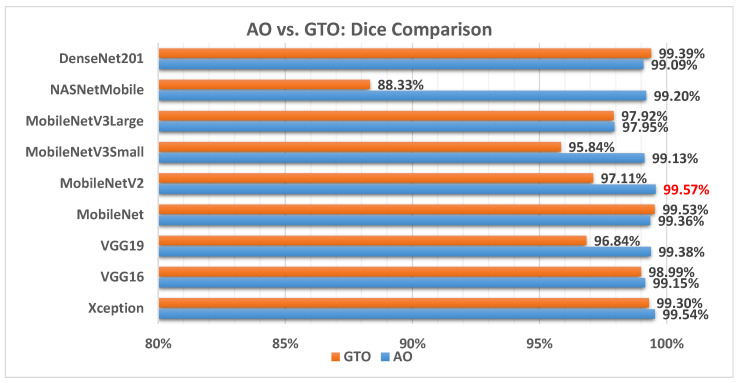
A comparison between the AO and GTO concerning the Dice coefficient.

Figure 11 illustrates a comparison between the performance of the AO and GTO with respect to the Dice coefficient. From the Dice point of view, the AO is better than the GTO in all models except for the MobileNet and DenseNet201 models. The best model is MobileNetV2.

**Figure 12:**
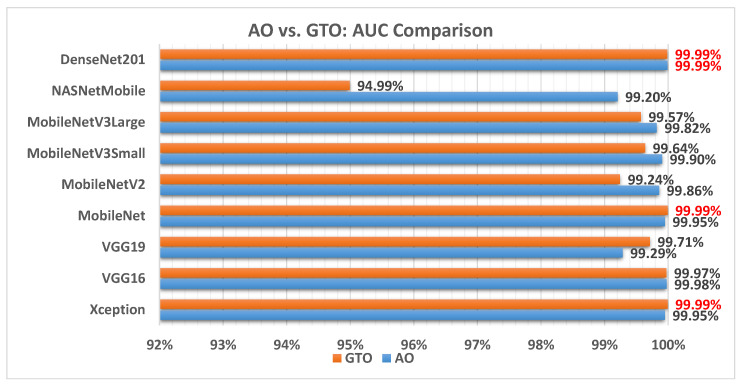
A comparison between the AO and GTO concerning the AUC.

Figure 12 illustrates a comparison between the performance of the AO and GTO with respect to AUC. From the AUC point of view, the AO is better than the GTO in all models except for the Xception, VGG16, and MobileNet models. The best models are Xception, MobileNet, and DenseNet201.

**Figure 13:**
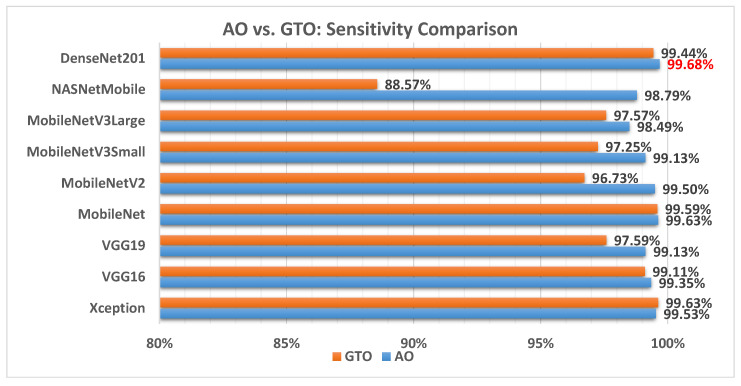
A comparison between the AO and GTO concerning the sensitivity.

Figure 13 illustrates a comparison between the performance of the AO and GTO with respect to sensitivity. From the sensitivity point of view, the AO is better than the GTO in all models except for the Xception model. The best model is DenseNet201.

**Figure 14:**
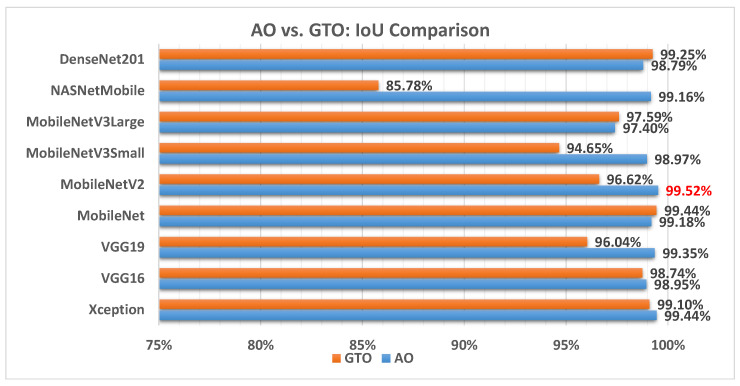
A comparison between the AO and GTO concerning the IoU.

Figure 14 illustrates a comparison between the performance of the AO and GTO with respect to IoU. From the IoU point of view, the AO is better than the GTO in all models except for the DenseNet201 and MobileNet models. The best model is MobileNetV2.

**Figure 15:**
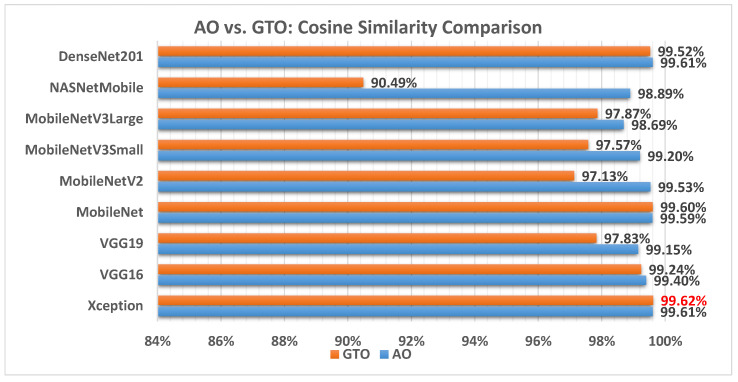
A comparison between the AO and GTO concerning the cosine similarity.

Figure 15 illustrates a comparison between the performance of the AO and GTO with respect to cosine similarity. From the cosine similarity point of view, the AO is better than the GTO in all models except for the Xception and MobileNet models. The best model is Xception.

**Figure 16:**
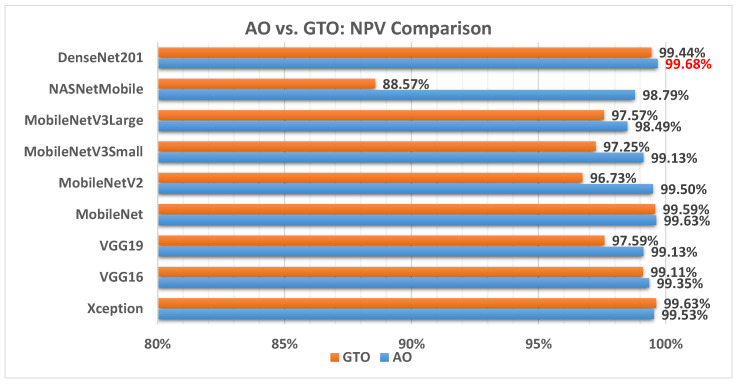
A comparison between the AO and GTO concerning the NPV.

Figure 16 illustrates a comparison between the performance of the AO and GTO with respect to NPV. From the NPV point of view, the AO is better than the GTO in all models except for the Xception and MobileNet models. The best model is DenseNet201.

**Figure 17:**
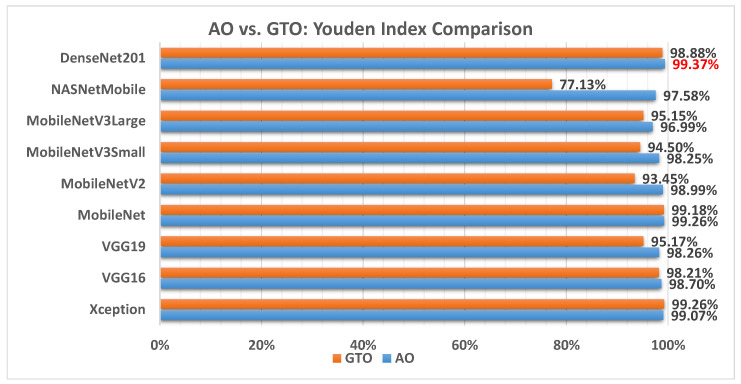
A comparison between the AO and GTO concerning the Youden index.

Figure 17 illustrates a comparison between the performance of the AO and GTO with respect to Youden index. From the Youden index point of view, the AO is better than the GTO in all models except for the Xception and MobileNet models. The best model is DenseNet201.

We can combine all of the performance metrics using the weighted sum approach so that the comparison is more statistical and promising. The weighted sum equation is shown in Equation (22).

(22) $$\begin{array}{c} \mathrm{WS}=\frac{1}{12}\times \mathrm{Accuracy}+\frac{1}{12}\times F1+\frac{1}{12}\times \mathrm{Precision}+\frac{1}{12}\times \mathrm{Recall}+\frac{1}{12}\times \mathrm{Specificity}+\frac{1}{12}\times \mathrm{AUC}\;+\\ \frac{1}{12}\times \mathrm{Sensitivity}+\frac{1}{12}\times \mathrm{IoU}+\frac{1}{12}\times \mathrm{Dice}+\frac{1}{12}\times \mathrm{Cosine}\;\mathrm{Similarity}+\frac{1}{12}\times \mathrm{Youden}\;\mathrm{Index}+\frac{1}{12}\times \mathrm{NPV} \end{array}$$

**Figure 18:**
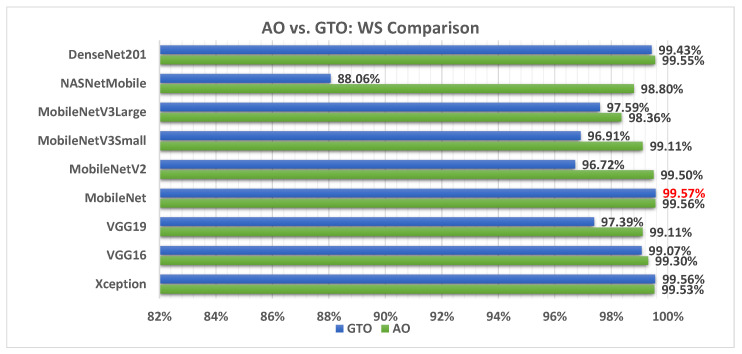
A comparison between the AO and GTO concerning the computed weight sum value.

Figure 18 illustrates a comparison between the performance of the AO and GTO with respect to the computed weight sum value. From the WS point of view, the AO is better than the GTO in all models except for the Xception and MobileNet models. However, the best model is the MobileNet produced by the GTO metaheuristic optimizer.

**Figure 19:**
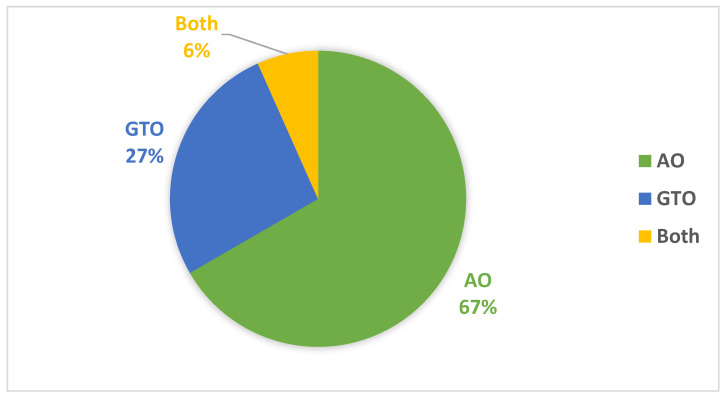
Graphical summary of the best models concerning each calculated performance metrics and the two metaheuristic optimizers.

Table 8 summarizes the best models concerning each calculated performance metric and the two metaheuristic optimizers. It shows that the AO outperforms the GTO with a ratio of 11 to 5. Figure 19 summarizes this graphically. The two metaheuristic optimizers, AO and GTO, were compared in terms of accuracy across different deep learning models as follows:

*AO results:*

- Average accuracy: 99.25%.
- Best model: DenseNet201.
- AO performed slightly better in accuracy, and DenseNet201 was recommended as the best model.

*GTO results:*

- Average accuracy: 97.27%.
- Best model: Xception.
- GTO achieved a lower average accuracy than AO, with Xception being the best-performing model recommended by GTO.

**Table 8:** Summary of the best models concerning each calculated performance metric and the two metaheuristic optimizers.

	Xception	VGG16	VGG19	MobileNet	MobileNetV2	MobileNetV3Small	MobileNetV3Large	NASNetMobile	DenseNet201
Accuracy									AO
F1 Score									AO
Precision									AO
Specificity									AO
AUC	GTO			GTO					Both
Sensitivity									AO
IoU					AO				
Cosine Similarity	GTO								
Youden Index									AO
NPV									AO
WS				GTO					

In Table 9, we see how the proposed method compares to similar research. This comparison demonstrates that the current study performs better than most similar investigations. The DenseNet201 model achieved an accuracy of 99.68% with the Aquila Optimizer (AO). The DenseNet201 model stands out in terms of accuracy and performance for several reasons such as dense connectivity, parameter efficiency, effective feature extraction, and fine-tuning through metaheuristic optimization. Thus, DenseNet201 is a top-performing model for oral cancer classification, leading to remarkable accuracy rates in this study.

The proposed framework contributes significantly to automating oral cancer detection through the following fundamental mechanisms:

- Metaheuristic optimization: The framework utilizes metaheuristic optimization algorithms, the AO and GTO, to automatically select and fine-tune various aspects of the deep learning model and preprocessing steps. This automation ensures that each specific model and dataset chooses the most effective hyperparameters, loss functions, and other settings. This reduces the need for manual trial-and-error tuning, saving time and effort.
- Transfer learning: The framework leverages pre-trained CNN models with “ImageNet” pre-trained weights. Transfer learning allows the models to learn relevant features from a large, diverse dataset (ImageNet) and adapt them for oral cancer classification. This knowledge transfer accelerates training and improves the model’s ability to extract meaningful features from histopathology slide images.
- Performance evaluation: The framework employs various performance metrics, including accuracy, AUC (area under the receiver operating characteristic curve), and specificity, to assess the model’s classification performance comprehensively. This automated evaluation allows for objective comparisons between different models and configurations.
- High accuracy: By automating the optimization process and leveraging the strengths of different deep learning models, the framework achieves high accuracy rates, with DenseNet201 being the most accurate model, reaching an average accuracy rate of 99.25% with AO.

In summary, the proposed framework streamlines and automates the complex process of deep learning model selection, hyperparameter tuning, and data preprocessing for oral cancer detection. This automation enhances the accuracy and efficiency of oral cancer diagnosis, potentially leading to earlier detection and improved patient outcomes.

## 6. Conclusions and Future Work

In this study, we have presented a novel and highly effective methodology for the classification of oral cancer utilizing pre-trained convolutional neural networks (CNNs) in conjunction with two distinct metaheuristic optimization algorithms, namely, the Gorilla Troops Optimizer (GTO) and Aquila Optimizer (AO). Our approach focuses on optimizing the preprocessing steps, selecting appropriate optimizers, and fine-tuning the hyperparameters of pre-trained CNNs. We conducted experiments on the Histopathologic Oral Cancer dataset obtained from Kaggle, which comprises two classes: “normal”, with 2494 images, and “OSCC”, with 2698 images. Our preprocessing pipeline involved resizing, dimension scaling, and balancing the datasets, followed by data augmentation to enhance model generalization.

The AO and GTO metaheuristic optimizers were instrumental in optimizing various transfer learning (TL) parameters, ensuring that each pre-trained CNN model reached its optimal configuration of hyperparameters. This approach, instead of random or grid searches, has demonstrated its reliability in producing superior results. We employed several key metrics to assess model performance, including accuracy, area under the curve (AUC), and specificity. Our study incorporated nine pre-trained CNN models, including NASNetMobile, Xception, VGG16, VGG19, DenseNet201, MobileNetV2, MobileNetV3Small, MobileNet, and MobileNetV3Large, all initialized with “ImageNet” pre-trained weights. The preliminary results showcase the effectiveness of our proposed framework, achieving an impressive average accuracy of 99.25% when the Aquila Optimizer uses the (recommended) KL divergence loss function for seven models. The GTO recommends the Poisson loss function for four models. Notably, the AO outperforms the GTO in terms of F1 score across all models except for Xception, with DenseNet201 emerging as the top-performing model.

In future research, we plan to rigorously assess and validate the comparison between the two optimizers using statistical tests such as Friedman’s and Wilcoxon rank-sum tests. Additionally, we envision extending the application of our framework to other domains, such as COVID-19 and breast cancer detection. Exploring diverse deep learning architectures and investigating the integration of swarm intelligence in oral cancer treatment are promising directions for our ongoing work.

## Figures and Tables

**Table 1:** The targeted different augmentation techniques and the corresponding configurations used to balance the datasets.

Technique	Value
Rotation	$30^{\circ }$
Width Shift Ratio	20%
Height Shift Ratio	20%
Shear Ratio	20%
Zoom Ratio	20%
Brightness Change	[0.8:1.2]
Vertical Flip	✔
Horizontal Flip	✔

**Table 2:** Indexing the solution with the definitions of hyperparameters.

Index	Definition
1	Loss function
2	The size of the batch
3	The dropout ratio
4	The TL learning ratio
5	Weights (i.e., parameters) optimizer
6	Dimension scaling technique
7	Apply DA or not
8	Rotation value (if DA is utilized)
9	Width shift value (if DA is utilized)
10	Height shift value (if DA is utilized)
11	Shear value (if DA is utilized)
12	Zoom value (if DA is utilized)
13	Horizontal flipping marker (if DA is utilized)
14	Vertical flipping marker (if DA is utilized)
15	Brightness changing range (if DA is utilized)

**Table 3:** Summary of experiment configurations.

Configuration	Specifications
Apply Dataset Shuffling?	Yes (random)
Input Image Size	(128×128×3)
Metaheuristic Optimizers	Aquila Optimizer (AO) and artificial Gorilla Troops Optimizer (GTO)
Train-To-Test Ratio	85% for training and validation, with 15% for testing
The Population Size	10
# Repetitions	10
# Epochs	5
Output Activation Function	Softmax
Pre-trained Models	Xception, VGG16, VGG19, MobileNet, MobileNetV2, MobileNetV3Small, MobileNetV3Large, NASNetMobile, and DenseNet201
Pre-trained Parameters Initializers	ImageNet
Losses Range	Categorical cross-entropy, categorical hinge, Poisson, squared hinge, KL divergence, and hinge
Parameter Optimizer Range	Adam, NAdam, AdaGrad, AdaDelta, SGD, SGD Nesterov, Ftrl, RMSProp Centered, AdaMax, RMSProp, and Adam AMSGrad
Dropout Range	[0→0.6]
Batch Size Span	4→48(step=4)
Pre-trained Model Learn Ratio Span	1→100(step=1)
Scaling Techniques	Normalize, standard, min–max, and max-abs
Apply DA?	[Yes,No]
DA Rotation Span	$0^{\circ }\to 45^{\circ }\;\left(\mathrm{step}=1^{\circ }\right)$
DA Width Shift Span	[0→0.25]
DA Height Shift Span	[0→0.25]
DA Shear Span	[0→0.25]
DA Zoom Span	[0→0.25]
DA Horizontal Flip Span	[Yes,No]
DA Vertical Flip Span	[Yes,No]
DA Brightness Span	[0.5→2.0]
Scripting Language	Python
Packages	Tensorflow, Keras, OpenCV, NumPy, and Matplotlib libraries
Working Environment	Google Colab equipped with a GPU (Intel(R) Xeon(R) CPU @ 2.00 GHz, Tesla T4 16 GB GPU, CUDA v.11.2, and 12 GB RAM)

**Table 4:** The reported best configurations concerning the used dataset and the AO metaheuristic optimizer.

Configuration	Xception	VGG16	VGG19	MobileNet	MobileNetV2	MobileNetV3Small	MobileNetV3Large	NASNetMobile	DenseNet201
Loss	KL Divergence	Categorical Cross-Entropy	Squared Hinge	KL Divergence	KL Divergence	KL Divergence	KL Divergence	KL Divergence	KL Divergence
Batch Size	24	8	12	28	40	24	20	28	24
Dropout	0.41	0.03	0.09	0.56	0.13	0.24	0.15	0.31	0.23
TL Learn Ratio	84	52	24	72	58	45	77	54	47
Optimizer	SGD Nesterov	AdaGrad	SGD Nesterov	AdaMax	AdaMax	AdaMax	SGD Nesterov	AdaMax	AdaGrad
Scaling Technique	Max-Abs	Standardization	Max-Abs	Max-Abs	Min–Max	Standardization	Standardization	Standardization	Min–Max
Apply Augmentation	Yes	Yes	Yes	No	Yes	Yes	Yes	Yes	No
Rotation Range	17	5	19	N/A	6	18	44	19	N/A
Width Shift Range	0.04	0.09	0.19	N/A	0.08	0.11	0.09	0.13	N/A
Height Shift Range	0.16	0.18	0.17	N/A	0.03	0.12	0.05	0.12	N/A
Shear Range	0.21	0.08	0.08	N/A	0.15	0.09	0.14	0.11	N/A
Zoom Range	0.07	0.16	0.02	N/A	0.14	0.1	0.1	0.13	N/A
Horizontal Flip	No	Yes	Yes	N/A	No	Yes	Yes	Yes	N/A
Vertical Flip	No	No	Yes	N/A	No	Yes	No	Yes	N/A
Brightness Range	0.57–1.44	0.74–0.81	0.71–1.37	N/A	1.01–1.13	1.06–1.14	0.84–1.77	1.14–1.22	N/A

**Table 5:** The reported results concerning the used dataset and the AO metaheuristic optimizer.

Configuration	Xception	VGG16	VGG19	MobileNet	MobileNetV2	MobileNetV3Small	MobileNetV3Large	NASNetMobile	DenseNet201
TP	5351	5357	5341	5356	5333	5329	5299	5311	5359
TN	5351	5357	5341	5356	5333	5329	5299	5311	5359
FP	25	35	47	20	27	47	81	65	17
FN	25	35	47	20	27	47	81	65	17
Accuracy	99.53%	99.35%	99.13%	99.63%	99.50%	99.13%	98.49%	98.79%	99.68%
F1 Score	99.53%	99.35%	99.13%	99.63%	99.50%	99.13%	98.49%	98.79%	99.68%
Precision	99.53%	99.35%	99.13%	99.63%	99.50%	99.13%	98.49%	98.79%	99.68%
Recall	99.53%	99.35%	99.13%	99.63%	99.50%	99.13%	98.49%	98.79%	99.68%
Specificity	99.53%	99.35%	99.13%	99.63%	99.50%	99.13%	98.49%	98.79%	99.68%
AUC	99.95%	99.98%	99.29%	99.95%	99.86%	99.90%	99.82%	99.20%	99.99%
Sensitivity	99.53%	99.35%	99.13%	99.63%	99.50%	99.13%	98.49%	98.79%	99.68%
IoU	99.44%	98.95%	99.35%	99.18%	99.52%	98.97%	97.40%	99.16%	98.79%
Dice	99.54%	99.15%	99.38%	99.36%	99.57%	99.13%	97.95%	99.20%	99.09%
Cosine Similarity	99.61%	99.40%	99.15%	99.59%	99.53%	99.20%	98.69%	98.89%	99.61%
Youden Index	99.07%	98.70%	98.26%	99.26%	98.99%	98.25%	96.99%	97.58%	99.37%
NPV	99.53%	99.35%	99.13%	99.63%	99.50%	99.13%	98.49%	98.79%	99.68%
Loss	0.016	0.020	0.518	0.017	0.022	0.028	0.049	0.109	0.017
FNR	0.005	0.006	0.009	0.004	0.005	0.009	0.015	0.012	0.003
FDR	0.005	0.006	0.009	0.004	0.005	0.009	0.015	0.012	0.003
Fallout	0.005	0.006	0.009	0.004	0.005	0.009	0.015	0.012	0.003
Categorical Cross-Entropy	0.016	0.020	0.184	0.017	0.022	0.028	0.049	0.147	0.017
Kullback–Leibler Divergence	0.016	0.020	0.108	0.017	0.022	0.028	0.049	0.109	0.017
Categorical Hinge	0.014	0.025	0.019	0.019	0.013	0.026	0.061	0.024	0.027
Hinge	0.507	0.513	0.509	0.510	0.506	0.513	0.531	0.512	0.514
Squared Hinge	0.511	0.518	0.518	0.514	0.511	0.520	0.543	0.523	0.517
Poisson	0.508	0.510	0.554	0.509	0.511	0.514	0.525	0.554	0.509
Logcosh Error	0.002	0.003	0.004	0.002	0.002	0.003	0.006	0.005	0.002
Mean Absolute Error	0.007	0.013	0.009	0.010	0.006	0.013	0.031	0.012	0.014
Mean IoU	0.491	0.359	0.882	0.520	0.842	0.497	0.256	0.943	0.257
Mean Squared Error	0.004	0.006	0.008	0.004	0.004	0.007	0.012	0.011	0.004
Mean Squared Logarithmic Error	0.002	0.003	0.004	0.002	0.002	0.004	0.006	0.005	0.002
Root Mean Squared Error	0.061	0.075	0.092	0.062	0.067	0.086	0.111	0.104	0.062

**Table 6:** The reported best configurations concerning the used dataset and the GTO metaheuristic optimizer.

Configuration	Xception	VGG16	VGG19	MobileNet	MobileNetV2	MobileNetV3Small	MobileNetV3Large	NASNetMobile	DenseNet201
MetaOptimizer	GTO	GTO	GTO	GTO	GTO	GTO	GTO	GTO	GTO
Loss	KL Divergence	Categorical Cross-Entropy	Poisson	KL Divergence	KL Divergence	Poisson	Poisson	Categorical Cross-Entropy	Poisson
Batch Size	48	44	12	36	32	20	40	4	36
Dropout	0.23	0.26	0.29	0.36	0.24	0.31	0.26	0	0.59
TL Learn Ratio	74	37	44	76	71	55	84	0	74
Optimizer	AdaMax	SGD Nesterov	AdaGrad	SGD	SGD	SGD Nesterov	AdaMax	AdaMax	SGD Nesterov
Scaling Technique	Min–Max	Standardization	Standardization	Min–Max	Standardization	Standardization	Standardization	Normalization	Standardization
Apply Augmentation	No	Yes	Yes	Yes	Yes	Yes	Yes	Yes	Yes
Rotation Range	N/A	28	23	21	13	37	34	0	12
Width Shift Range	N/A	0.24	0.16	0.18	0.14	0.16	0.15	0	0.23
Height Shift Range	N/A	0.13	0.15	0.11	0.15	0.17	0.08	0	0.16
Shear Range	N/A	0.1	0.08	0.03	0.05	0.02	0	0	0.17
Zoom Range	N/A	0.08	0.17	0.12	0.13	0.08	0.15	0	0.11
Horizontal Flip	N/A	No	No	No	No	Yes	Yes	Yes	No
Vertical Flip	N/A	No	No	Yes	No	Yes	Yes	Yes	Yes
Brightness Range	N/A	0.64–0.82	1.0–1.12	1.01–1.65	0.99–1.4	0.8–1.26	1.08–1.68	0.5–0.5	0.53–0.81

**Table 7:** The reported results concerning the used dataset and the GTO metaheuristic optimizer.

Configuration	Xception	VGG16	VGG19	MobileNet	MobileNetV2	MobileNetV3Small	MobileNetV3Large	NASNetMobile	DenseNet201
TP	5356	5320	5258	5342	5200	5232	5230	4779	5334
TN	5356	5320	5258	5342	5200	5232	5230	4779	5334
FP	20	48	130	22	176	148	130	617	30
FN	20	48	130	22	176	148	130	617	30
Accuracy	99.63%	99.11%	97.59%	99.59%	96.73%	97.25%	97.57%	88.57%	99.44%
F1 Score	99.63%	99.11%	97.59%	99.59%	96.73%	97.25%	97.57%	88.57%	99.44%
Precision	99.63%	99.11%	97.59%	99.59%	96.73%	97.25%	97.57%	88.57%	99.44%
Recall	99.63%	99.11%	97.59%	99.59%	96.73%	97.25%	97.57%	88.57%	99.44%
Specificity	99.63%	99.11%	97.59%	99.59%	96.73%	97.25%	97.57%	88.57%	99.44%
AUC	99.99%	99.97%	99.71%	99.99%	99.24%	99.64%	99.57%	94.99%	99.99%
Sensitivity	99.63%	99.11%	97.59%	99.59%	96.73%	97.25%	97.57%	88.57%	99.44%
IoU	99.10%	98.74%	96.04%	99.44%	96.62%	94.65%	97.59%	85.78%	99.25%
Dice	99.30%	98.99%	96.84%	99.53%	97.11%	95.84%	97.92%	88.33%	99.39%
Cosine Similarity	99.62%	99.24%	97.83%	99.60%	97.13%	97.57%	97.87%	90.49%	99.52%
Youden Index	99.26%	98.21%	95.17%	99.18%	93.45%	94.50%	95.15%	77.13%	98.88%
NPV	99.63%	99.11%	97.59%	99.59%	96.73%	97.25%	97.57%	88.57%	99.44%
Loss	0.014	0.022	0.535	0.013	0.101	0.543	0.537	0.287	0.508
FNR	0.004	0.009	0.024	0.004	0.033	0.028	0.024	0.114	0.006
FDR	0.004	0.009	0.024	0.004	0.033	0.028	0.024	0.114	0.006
Fallout	0.004	0.009	0.024	0.004	0.033	0.028	0.024	0.114	0.006
Categorical Cross-Entropy	0.014	0.022	0.070	0.013	0.101	0.086	0.074	0.287	0.015
Kullback–Leibler Divergence	0.014	0.022	0.070	0.013	0.101	0.086	0.074	0.287	0.015
Categorical Hinge	0.021	0.030	0.095	0.014	0.087	0.125	0.062	0.350	0.018
Hinge	0.510	0.515	0.547	0.507	0.543	0.562	0.531	0.675	0.509
Squared Hinge	0.514	0.522	0.567	0.511	0.570	0.585	0.551	0.762	0.514
Poisson	0.507	0.511	0.535	0.506	0.550	0.543	0.537	0.643	0.508
Logcosh Error	0.002	0.003	0.009	0.002	0.012	0.011	0.009	0.040	0.002
Mean Absolute Error	0.010	0.015	0.047	0.007	0.043	0.062	0.031	0.175	0.009
Mean IoU	0.373	0.390	0.253	0.471	0.332	0.250	0.425	0.250	0.463
Mean Squared Error	0.004	0.007	0.020	0.004	0.027	0.023	0.020	0.087	0.004
Mean Squared Logarithmic Error	0.002	0.003	0.010	0.002	0.013	0.011	0.010	0.043	0.002
Root Mean Squared Error	0.060	0.082	0.141	0.061	0.163	0.150	0.141	0.294	0.066

**Table 9:** Comparison between the suggested approach and related studies.

Study	Year	Dataset	Approach	Best Accuracy
Aubreville et al. [40]	2017	Confocal Laser Endomicroscopy (CLE)	CNN	88.3%
Jeyaraj et al. [14]	2019	N/A	CNN	91.4%
Ariji et al. [41]	2019	CT	CNN	78.2%
Bhandari et al. [50]	2020	MRI	CNN	94.5%
Current Study	2022	Histopathological	Hybrid	99.68%

## Data Availability

The data that support the findings of this study are available on request.
